# Serum Levels of Persistent Organic Pollutants and Insulin Secretion among Children Age 7–9 Years: A Prospective Cohort Study

**DOI:** 10.1289/EHP147

**Published:** 2016-06-07

**Authors:** Su Hyun Park, Eunhee Ha, Young Sun Hong, Hyesook Park

**Affiliations:** 1 Department of Preventive Medicine,; 2 Department of Occupational and Environmental Medicine, and; 3 Department of Internal Medicine, School of Medicine, Ewha Womans University, Seoul, Korea

## Abstract

**Background:**

Persistent organic pollutants (POPs) are endocrine disruptors and have been suggested as possible risk factors for diabetes. Few studies have been performed to investigate this association among children.

**Objectives:**

In this study, we prospectively examined the relationship between the serum concentration of POPs and glucose metabolism in children.

**Methods:**

Data were collected from the Ewha Birth & Growth Cohort Study, an ongoing birth cohort study initially constructed between 2001 and 2006. In 2010–2012, the POP concentration was measured in serum from a total of 214 children, 7–9 years of age. Using fasting glucose and insulin measurements at both baseline and the second year of follow-up, the homeostatic model assessment of beta-cell function (HOMA-β) and homeostatic model assessment of insulin resistance (HOMA-IR) were calculated. Multiple linear regression analysis and a linear mixed-effects model were used to determine the relationship between POP tertiles and metabolic biomarkers.

**Results:**

Compared with the lowest tertile of total marker PCBs, participants in the third tertile had decreased HOMA-β values, after adjustment for age, sex, body mass index z-score, mother’s education, ponderal index, and history of breastfeeding (–18.94%; 95% CI: –32.97%, –1.98%). In a linear mixed model, the HOMA-β values were still lower in subjects in the highest compared with the lowest tertile of total PCBs at the 2-year follow-up period (108.3 vs. 135.0, respectively).

**Conclusion:**

The results of the study suggested that exposure to POPs among children might affect insulin secretory function, which could lead to an increased risk of developing diabetes.

**Citation:**

Park SH, Ha EH, Hong YS, Park H. 2016. Serum levels of persistent organic pollutants and insulin secretion among children age 7–9 years: a prospective cohort study. Environ Health Perspect 124:1924–1930; http://dx.doi.org/10.1289/EHP147

## Introduction

Persistent organic pollutants (POPs), including organochlorine pesticides (OCPs) and polychlorinated biphenyls (PCBs), are present in the environment due to their characteristics of persistency and bioaccumulation, although their usage and production have been banned and restricted since the 1970s (UNEP 2003). Human exposure to POPs occurs primarily through consumption of contaminated fish, meat, and dairy food products in the general population (Polder et al. 2010; WHO 2014). The well-known possible health outcomes for children exposed to POPs include delays in cognitive development (Boersma and Lanting 2000; Rogan and Gladen 1991; Stewart et al. 2003), pubertal development (Özen and Darcan 2011), and behavioral problems (Lai et al. 2002; Tatsuta et al. 2012).

In recent years, epidemiologic evidence has emerged suggesting that exposure to environmental endocrine-disrupting chemicals might interfere with normal physiologic processes, resulting in an increased risk of diabetes. The pathogenesis of type 2 diabetes mellitus (T2DM) involves impairments in insulin resistance and secretion, which stems from both genetic and environmental causes. Various studies in adults have suggested that exposure to POPs is associated with T2DM (Airaksinen et al. 2011; Al-Othman et al. 2014; Lee et al. 2011; MacNeil et al. 2009; Philibert et al. 2009; Tang et al. 2014; Taylor et al. 2013; Turyk et al. 2009, 2015; Wu et al. 2013). Several possible underlying mechanisms and/or pathways linking POPs to T2DM have been suggested, including potential involvement of POPs in insulin secretion or sensitivity; however, the complete picture is not yet understood fully.

Children are more susceptible to environmental toxins than are adults due to their behavioral and physiological characteristics; however, few studies have been conducted in young children. A life-course approach is considered to be a key in identifying associations between early-life exposure and later health outcomes, suggesting the importance of studying children as a basis for future analysis (WHO and International Longevity Centre–UK 2000). Only one cross-sectional study (Jensen et al. 2014) investigated the relationship between PCBs and the indicators of glucose metabolism in healthy children.

In this study, we measured the serum concentration of POPs in children 7–9 years of age and investigated how it may affect metabolic biomarkers, possibly leading to diabetes risks later in life, by conducting a prospective cohort study.

## Methods

### Study Population

In this prospective cohort study, 214 children 7–9 years of age were selected from participants in the Ewha Birth & Growth Study, a prospective hospital-based birth cohort. A detailed description of the Ewha Birth & Growth Cohort Study is given by Park et al. (2009). Briefly, a total of 940 pregnant women were recruited at their first prenatal care visits, during weeks 24–28 of pregnancy, at Ewha Womans University Mokdong Hospital, Seoul, Korea. The recruitment period was from 2001 to 2006. Study follow-ups have been conducted annually since 2005. In 2010–2012, there were 330 children who reached the required age (7–9 years) and participated in at least one of the follow-up assessments. From the 330 eligible children, 214 subjects (64.8%) had available blood levels for POP analyses. We compared metabolic biomarkers, including body mass index (BMI), glucose, insulin, triglyceride, and high-density lipoprotein (HDL), among children 7–9 years of age who participated in the follow-up assessment in 2012 to explore differences in the characteristics of study subjects and non-study subjects (see Table S1). As shown in Table S1, there were no significant differences between study and non-study subjects. The follow-up program included self-administered sociodemographic questionnaires (parental income and education level) and the collection of blood and urine samples after fasting for at least 9–12 hr. All subjects and parents provided written informed consent to participate in the study. The sample size was calculated using G*power software (version 3.1.9.2) (Faul et al. 2007), considering the possibility of follow-up loss and the high participation and response rates in the study. The estimated sample sizes were 203 subjects to achieve 99% power and 153 subjects to yield 95% power, with an alpha level of 0.05 and an effect size of 0.15. A total of 214 children who participated from 2010 to 2012 were selected for this study, and a total of 82 children were eliminated after failing to follow-up after 2 years (38.3%). The study protocol was approved by the Institutional Review Board of Ewha Womans University Hospital.

In 2010 and 2012, blood samples were collected from children who had fasted for 8 hr. Peripheral venous blood samples (2 mL) were collected from the participants. Serum was separated from peripheral venous blood and stored frozen at –70°C until required for analyses. POPs concentrations and metabolic biomarkers, including fasting glucose and insulin levels were measured simultaneously at the baseline. Only metabolic biomarkers (outcome variables) were measured twice (at baseline and 2-year follow-up).

### Analyses of POPs

A total of 51 POPs, including 19 OCPs [oxychlordane, *trans*-nonachlor, *cis*-nonachlor, heptachlor, *trans*-heptachlorepoxide, *cis*-heptachlorepoxide, hexachlorobenzene, *trans*-chlordane, *cis*-chlordane, α-, β-, γ-isomers of hexachlorocyclohexane (HCH), *p*,*p*´-DDE (dichlorodiphenyldichloroethylene), *o*,*p*´-DDT (dichlorodiphenyltrichloroethane), *p*,*p*´-DDD (dichlorodiphenyldichloroethane), *o*,*p*´-DDD *p*,*p*´-DDE, and *o*,*p*´-DDE] and 32 PCB congeners (IUPAC nos: 1, 3, 4, 15, 19, 28, 37, 52, 54, 77, 81, 101, 104, 105, 114, 118, 123, 126, 138, 153, 155, 156, 157, 167, 169, 180, 188, 189, 202, 205, 206, and 208) were measured at baseline by the isotope dilution method using gas chromatography high resolution mass spectrometry (GC-HRMS) at the laboratory of Labfrontier (Seoul, Korea). Extraction and sample clean up procedures were based on a method used by the U.S. Centers for Disease Control and Prevention (CDC 2006), with some modifications (CDC 2006).

In brief, serum samples were spiked with isotopically labeled OCP standards (ES-5400; Cambridge Isotope Labs., Tewksbury, MA, USA) and isotopically labeled PCB standards (68C-LCS; Wellington Labs, Guelph, ON, Canada). Extraction was performed using C18 solid-phase extraction (SPE) cartridges (Waters, Dublin, Ireland). The eluate was applied to a silica gel/florisil SPE cartridge (Waters) and then eluted with 16 mL dichloromethane/hexane (1:1 vol/vol). GC-HRMS measurements were made on a JMS-800D (JEOL, Tokyo, Japan) using a 6890N gas chromatograph (Agilent Technologies, Santa Clara, CA, USA) and a DB-5MS column (60 m × 25 mm × 0.25 μm).

For quality control (QC) and quality assurance (QA), serum samples were incorporated in each batch of 15 samples. The recovery of internal standards ranged from 50% to 120%, which was considered satisfactory. The relative standard deviation of QC/QA samples was below 15% for all compounds with values above the limit of detection (LOD) in the QA/QC samples.

### Measurement of Anthropometric and Metabolic Parameters

Anthropometric data were collected by trained nurses and medical students. Height and weight were measured while the children were wearing light clothing without shoes, with a stadiometer and calibrated scale (DS-102; Dong Sahn Jenix, Co., Ltd., Seoul, Korea). The BMI was calculated as weight divided by height squared (kg/m^2^). Due to the skewness of the BMI distribution, the *z*-score of the BMI was calculated using the Stata command zanthro, which used the 2000 U.S. growth charts as the reference distribution (Kuczmarski et al. 2000). The ponderal index (PI) at birth was calculated as follows: birth weight (kg)/ birth length (m^3^).

Fasting insulin, fasting glucose, homeostatic model assessment of insulin resistance (HOMA-IR), and homeostatic model assessment of beta-cell function (HOMA-β) were used as metabolic biomarkers. The HOMA model presents glucose-insulin homeostasis and predicts glucose uptake and insulin production. It has been used as a mathematical estimate that accounts for the interrelationship between insulin sensitivity and pancreatic β-cell function in a feedback loop. Although this model is not a gold standard method for measuring insulin sensitivity, it has been used widely for assessing insulin resistance and β-cell function in epidemiological studies of diabetes (Matthews et al. 1985; Wallace et al. 2004). Fasting insulin was measured in blood samples after at least an 8-hr fast, using an immunoradiometric assay kit (Biosource Europe, Nivelles, Belgium). Fasting glucose was measured using an automatic analyzer (model 7180; Hiyachi, Tokyo, Japan). HOMA-IR was calculated as follows: fasting insulin (mU/mL) × fasting glucose (mg/dL)/405. HOMA-β was calculated using the following formula: 360 × fasting insulin (mU/mL)/[fasting glucose (mg/dL) – 63].

### Statistical Analyses

Concentrations of all metabolic biomarkers and POPs measured were log-transformed to control for the skewed distribution. For all statistical analyses, levels below the LOD were entered into the data set as 50% of the LOD. We summed the individual PCBs and OCPs to investigate whether the mixture of compounds affected glucose metabolism. “Total OCPs” was defined as the sum of 19 measured OCPs [oxychlordane, *trans*-nonachlor, *cis*-nonachlor, heptachlor, *trans*-heptachlorepoxide, *cis*-heptachlorepoxide, hexachlorobenzene (HCB), *trans*-chlordane, *cis*-chlordane, α-, β-, γ-isomers of HCH, *p*,*p*´-DDE, *o*,*p*´-DDT, *p*,*p*´-DDD, *o*,*p*´-DDD, *p*,*p*´-DDE, and *o*,*p*´-DDE]. “Total PCBs” was defined as the sum of 32 measured PCBs (IUPAC nos: 1, 3, 4, 15, 19, 28, 37, 52, 54, 77, 81, 101, 104, 105, 114, 118, 123, 126, 138, 153, 155, 156, 157, 167, 169, 180, 188, 189, 202, 205, 206, and 208). “Total marker PCBs,” which comprised approximately half of the total non-dioxin-like PCBs and had comparatively high detection rates, was defined as the sum of six PCB congeners: PCBs 28, 52, 101, 118, 138, and 153. Serum PCBs and OCPs were divided into tertiles, with the lowest tertile serving as the referent.

Due to the high concentration of POPs in the lipid component of the blood, lipid-adjusted concentrations (ng/g lipid) were calculated using the formula proposed by Phillips et al. (1989) and confirmed by Bernert et al. (2007): total lipids (mg/dL) = 2.27 × total cholesterol + triglycerides + 62.3.

Means and standard deviations (SD) of the metabolic biomarkers were calculated according to the POP concentration tertiles (T1–T3). Covariates included baseline age, sex, BMI *z*-score, mother’s education level, PI value, and history of breastfeeding (yes/no). We included the breastfeeding information for multiple linear regression and linear mixed-effects models as a covariate, because total OCP and PCB concentrations were higher in children who were breastfed than in children who were not breastfed (78.57 vs. 46.16 for total OCPs; 25.55 vs. 20.10 for total PCBs) (data not shown). Breastfeeding was also considered to be a possible confounder in previous studies (Jensen et al. 2014; Lam et al. 2013). The ages of the children were recorded at the time of measuring POPs. The follow-up measurements were made 2 years later at a fairly uniform interval (the follow-up duration was roughly equivalent); therefore, only the baseline age was included as a covariate in the models.

In this study, we conducted a cross-sectional analysis to determine the association between exposure to POPs and metabolic biomarkers, such as glucose, insulin, HOMA-β, and HOMA-IR at baseline. Multiple linear regression analysis was conducted to examine the association between POP concentrations and metabolic biomarkers, with an adjustment for sex, baseline age, PI, mother’s education, and BMI *z*-score. We included POP concentrations as a categorical exposure variable (tertiles) and metabolic biomarkers as a continuous outcome variable.

To establish temporality, we performed a longitudinal analysis to estimate the association between repeated measures of HOMA-β, as the outcome, and a single baseline measure of POP concentrations, as the exposure. We assumed that POP concentrations remained fairly constant throughout the study period (2 years) in this study (Park et al. 2016b), because we focused on establishing the temporality of the relationships so that the exposure preceded the outcome. A linear mixed-effects model (PROC MIXED; SAS Institute Inc.) was used to determine whether baseline levels of POP affected HOMA-β values at the 2-year follow-up. To take into account the repeated observations within subjects, the model was fitted to select an appropriate working correlation structure. A first-order autoregressive [AR(1)] structure was selected for the final model.

We used a significance level of α = 0.05 in all analyses. The statistical analyses were conducted using STATA version 12.0 (StataCorp, College Station, TX, USA) and SAS version 9.3 (SAS Institute Inc., Cary, NC, USA).

## Results

### Study Subjects

Table 1 presents the baseline descriptive characteristics of the study population. Among the 214 children, 106 (49.5%) were male. The mean age in months of the children at baseline was 90.1 ± 9.5. The mean HOMA-IR and HOMA-β values were 1.6 ± 0.7 and 189.37 ± 6.85, respectively. Approximately 80% of mothers had more than a high school education (78.7%).

**Table 1:** Baseline characteristics of the children participating in the Ewha Birth & Growth Cohort Study.

Characteristic	*n *(%) or mean ± SD	(95% CI)
Male	106 (49.5)
Age (month)	90.07 ± 9.50	(88.80, 91.35)
Birth weight (kg)	3.18 ± 0.57	(3.11, 3.26)
Birth length (cm)	49.03 ± 2.51	(48.69, 49.37)
Ponderal index (kg/m^3^)^*a*^	26.62 ± 3.21	(26.19, 27.06)
Current weight (kg)	28.19 ± 6.04	(27.38, 29.01)
BMI (kg/m^2^)^*b*^	16.67 ± 2.73	(16.30, 17.04)
BMI *z*-score^*c*^	0.29 ± 1.07	(0.15, 0.43)
Glucose (mg/dL)	79.50 ± 0.53	(78.46, 80.55)
Insulin (μIU/mL)	8.14 ± 0.22	(7.71, 8.58)
HOMA-IR^*d*^	1.61 ± 0.65	(1.52, 1.69)
HOMA-β%^*e*^	189.37 ± 6.85	(175.85, 202.87)
Mother’s education level
≤ High school graduate	45 (21.3)
College graduate	147 (69.7)
Postgraduate	19 (9.0)
Values are means ± SD for continuous variables and *n *(%) for categorical variables. ^***a***^Weight (kg)/length (m^3^). ^***b***^Weight (kg)/height (m^2^). ^***c***^Kuczmarski et al. (2000). ^***d***^HOMA-IR was calculated as follows: fasting insulin (mU/mL) × fasting glucose (mg/dL))/405. ^***e***^HOMA-β was calculated as follows: 360 × fasting insulin (mU/mL)/[fasting glucose (mg/dL) – 63].		

### Associations of POPs with Metabolic Biomarkers

The mean differences in biomarkers at baseline among POP concentration tertiles were compared (Table 2). Subjects in the highest tertile had a higher level of glucose (78.10 vs. 81.33 mg/dL for total marker PCBs) and a lower HOMA-β value (207.49 vs. 155.72 for total marker PCBs). Multiple linear regression models were used to analyze the association between metabolic biomarkers (log-transformed HOMA-β, HOMA-IR, glucose, and insulin) at baseline and POP concentrations (Table 3). Overall, serum POP concentrations were associated negatively with HOMA-β, positively with glucose, and negatively with insulin, whereas no relationship was observed with HOMA-IR. The HOMA-β value decreased by –18.94% at a higher total marker PCB concentration tertile [95% confidence interval (CI): –32.97, –1.98].

**Table 2:** Means and standard deviations of metabolic biomarkers*^a^* according to POP concentration tertile.

Compound^*b*^ (ng/g lipid)	Glucose		Insulin		HOMA-β		HOMA-IR	
	Mean	Std	Mean	Std	Mean	Std	Mean	Std
PCB-138
≤ 1.76	78.35	8.22	8.92	3.00	230.06	117.92	1.73	0.61
1.761–3.06	79.49	8.05	8.27	4.26	176.03	67.05	1.64	0.83
> 3.07	80.68	6.84	7.24	1.80	162.00	89.47	1.45	0.41
PCB-153
≤ 3.05	78.41	8.22	9.12	4.42	217.07	106.54	1.77	0.83
3.06–6.06	79.35	8.01	7.77	2.68	186.87	96.56	1.54	0.59
> 6.06	80.76	6.86	7.55	1.95	164.60	83.58	1.51	0.43
PCB-180
≤ 1.82	79.01	7.23	8.50	2.62	208.98	96.57	1.66	0.54
1.888–4.21	79.01	8.46	8.33	4.58	189.85	101.46	1.64	0.88
> 4.22	80.49	7.51	7.61	1.88	168.68	92.61	1.51	0.41
Total PCBs^*c*^
≤ 18.73	77.91	7.96	8.57	4.44	207.20	94.28	1.65	0.83
19.17–31.73	79.92	6.63	8.12	2.79	198.91	110.83	1.61	0.60
> 31.74	80.59	8.54	7.73	2.12	162.23	82.94	1.54	0.48
Marker PCBs^*d*^
≤ 12.44	78.32	8.48	8.83	4.41	215.66	107.88	1.71	0.83
12.45–21.83	79.18	6.91	7.96	2.87	197.42	106.84	1.57	0.61
> 21.83	81.03	7.62	7.63	1.84	154.39	62.20	1.54	0.43
β-HCH
≤ 4.36	78.41	8.87	8.33	2.73	214.14	105.36	1.62	0.58
4.37–9.00	79.68	7.41	8.49	4.39	186.77	97.51	1.67	0.83
> 9.00	80.42	6.81	7.61	2.16	167.47	85.60	1.52	0.46
*p,p*’-DDE
≤ 31.45	78.73	8.82	9.17	4.38	214.23	108.51	1.78	0.82
31.46–61.03	79.94	7.62	7.67	2.32	188.16	99.88	1.53	0.53
> 61.03	79.83	6.73	7.61	2.42	166.16	78.24	1.50	0.50
*trans*-Nonachlor
≤ 0.45	77.86	8.54	8.56	4.21	212.67	112.61	1.65	0.79
0.90–1.54	80.20	7.03	8.37	2.83	192.86	88.78	1.67	0.61
> 1.54	80.85	7.08	7.47	1.98	160.43	79.65	1.50	0.44
Total OCPs^*e*^
≤ 52.54	78.87	8.96	9.03	4.38	209.06	103.15	1.76	0.82
53.84–96.62	79.10	7.19	7.94	2.52	203.71	107.59	1.56	0.55
> 96.62	80.46	7.11	7.46	2.37	155.99	73.17	1.49	0.51
Note: *p,p*’-DDE, *p,p*’-dichlorodiphenyldichloroethylene; HCB, hexachlorobenzene; β-HCH, β-hexachlorocyclohexane; HOMA-β, homeostatic model assessment of beta-cell function; HOMA-IR, homeostatic model assessment of insulin resistance; OCP, organochlorine pesticides; PCB, polychlorinated biphenyls. ^***a***^Log transformed. ^***b***^The limit of detection was set as LOD/2. ^***c***^Sum of all 32 PCBs measured. ^***d***^Sum of marker PCBs (PCBs 28, 52, 101, 138, 153, and 180). ^***e***^Sum of all measured 19 OCPs.								

**Table 3:** Adjusted percentage change*^a^* in metabolic biomarkers*^b^* according to POP concentration tertile (exposure, ng/g lipid).

Compound^*c*^ (ng/g lipid)	Glucose [% change (95% CI)]	Insulin [% change (95% CI)]	HOMA-β [% change (95% CI)]	HOMA-IR [% change (95% CI)]
PCB-138
≤ 1.76	Referent			
1.761–3.06	2.02 (–1.98, 6.18)	1.01 (–8.61, 11.63)	–3.92 (–21.34, 17.35)	3.05 (–8.61, 15.03)
> 3.07	3.05 (–1.00, 7.25)	–5.82 (–15.63, 4.08)	–14.79 (–30.23, 4.08)	–2.96 (–13.93, 9.42)
PCB-153
≤ 3.05
3.06–6.06	1.01 (–2.96, 4.08)	–9.52* (–18.13, –0.20)	–9.52 (–25.92, 0.90)	–9.52 (–18.94, 2.02)
> 6.06	2.02 (–1.98, 6.18)	–10.42* (–19.75, –1.00)	–14.79 (–30.23, 4.08)	–9.52 (–18.94, 2.02)
PCB-180
≤ 1.82
1.888–4.21	0.30 (–3.92, 4.08)	–1.98 (–11.31, 9.42)	–1.00 (–18.94, 20.92)	–1.00 (–12.19, 10.52)
> 4.22	1.01 (–2.96, 5.13)	–5.82 (–15.63, 5.13)	–6.76 (–24.42, 15.03)	–3.92 (–15.63, 8.33)
Total PCBs^*d*^
≤ 18.73
19.17–31.73	2.02 (–1.00, 6.18)	–2.96 (–12.19, 7.25)	–8.61 (–24.42, 10.52)	–1.00 (–11.31, 10.52)
> 31.74	1.01 (–2.96, 5.13)	–11.31* (–19.75, –1.98)	–17.30 (–32.29, 1.01)	–10.42 (–20.55, 0.30)
Marker PCBs^*e*^
≤ 12.44
12.45–21.83	1.01 (–2.96, 4.08)	–5.82 (–14.79, 4.08)	–7.69 (–23.66, 11.63)	–4.88 (–14.79, 6.18)
> 21.83	2.02 (–1.98, 5.13)	–9.52 (–18.13, 0.20)	–18.94* (–32.97, –1.98)	–7.69 (–18.13, 3.05)
β-HCH
≤ 4.36
4.37–9.00	1.01 (–1.98, 5.13)	1.01 (–8.61, 11.63)	1.01 (–17.30, 22.14)	3.05 (–8.61, 15.03)
> 9.00	3.05 (–1.00, 7.25)	–4.88 (–13.93, 6.18)	–22.12* (–36.24, –4.88)	–1.98 (–13.06, 10.52)
*p,p*’-DDE
≤ 31.45
31.46–61.03	2.02 (–1.98, 6.18)	–3.92 (–13.06, 6.18)	–13.93 (–28.82, 5.13)	–1.98 (–13.06, 10.52)
> 61.03	1.01 (–1.98, 5.13)	–6.76 (–16.47, 3.05)	–16.47 (–31.61, 1.01)	–5.82 (–16.47, 6.18)
*trans*-Nonachlor
≤ 0.45
0.90–1.54	2.02 (–1.00, 6.18)	2.02 (–7.69, 12.75)	–1.98 (–18.94, 18.53)	4.08 (–6.76, 17.35)
> 1.54	4.08* (1.01, 8.33)	–1.98 (–11.31, 9.33)	–25.92** (–38.74, –10.42)	3.05 (–8.61, 15.03)
Total OCPs^*f*^
≤ 52.54
53.84–96.62	–0.40 (–3.92, 3.05)	–1.00 (–10.42, 9.42)	–0.10 (–17.30, 20.92)	–1.00 (–12.19, 10.52)
> 96.62	0.30 (–2.96, 4.08)	–8.61 (–17.30, 1.01)	–13.93 (–28.82, 4.08)	–8.61 (–18.13, 3.05)
Note: *p,p*’-DDE, *p,p*’-dichlorodiphenyldichloroethylene; HCB, hexachlorobenzene; β-HCH, β-hexachlorocyclohexane; HOMA-β, homeostatic model assessment of beta-cell function; HOMA-IR, homeostatic model assessment of insulin resistance; OCP, organochlorine pesticides; PCB, polychlorinated biphenyls. ^***a***^Adjusted percentage change = [exp(β) – 1] × 100. ^***b***^Log transformed. ^***c***^The limit of detection was set as LOD/2. ^***d***^Sum of all measured 32 PCBs. ^***e***^Sum of marker PCBs (PCBs 28, 52, 101, 138, 153, and 180). ^***f***^Sum of all measured 19 OCPs models were adjusted for baseline age (months), sex, WHO BMI *z*-score, mother’s education level, ponderal index, and breastfeeding (yes/no). *p < 0.05. **p < 0.01.				

Among the OCPs, the higher tertiles of β–HCH and *trans*-nonachlor showed a significant negative association with the HOMA-β value. The HOMA-β value diminished by –22.12% at the highest β-HCH concentration tertile (95% CI: –36.24, –4.88), whereas no significant relationships were found for the glucose and insulin levels and HOMA-IR value. The HOMA-β value was reduced by –25.92%, according to the *trans*-nonachlor tertiles from the lowest to the highest tertile (95% CI: –38.74, –10.42) (Table 3).

**Figure 1:**
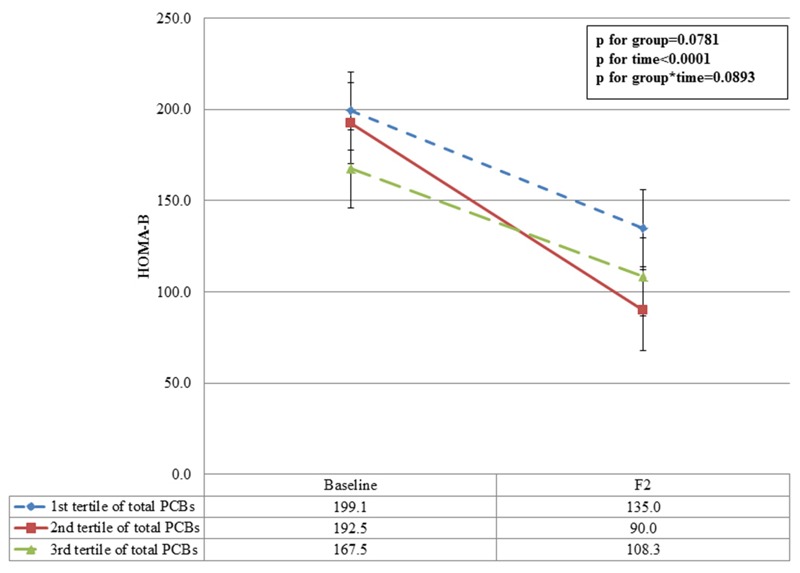
Adjusted least square means of HOMA-β values at baseline and the 2-year follow-up according to tertiles of total PCBs. F2: 2 year follow-up. Adjusted for baseline age (months), sex, BMI *z*-score, mother’s education level, ponderal index, and breastfeeding (yes/no).

**Figure 2:**
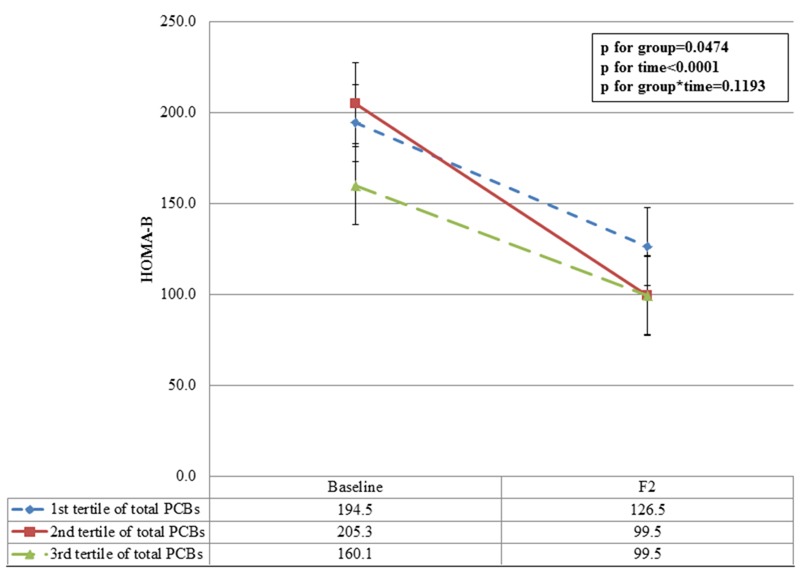
Adjusted least square means of HOMA-β values at baseline and the 2-year follow-up according to tertiles of total OCPs. F2: 2 year follow-up. Adjusted for baseline age (months), sex, BMI *z*-score, mother’s education level, ponderal index, and breastfeeding (yes/no).

We used a linear mixed-effects model with longitudinal data to investigate the association between baseline POP concentrations and HOMA-β at baseline and 2 years later. The trends were similar to the baseline estimates; subjects in the higher tertiles had lower HOMA-β values at the 2-year follow-up. The least squares means (with 95% CIs) of HOMA-β according to the tertiles of total PCBs and total OCPs are shown in Figures 1 and 2. Subjects in the highest, compared with the lowest, total PCB tertile had lower HOMA-β values at baseline after covariate adjustments (167.5 vs. 199.1, respectively). The HOMA-β values remained lower in subjects in the highest versus lowest total PCB tertile after covariate adjustments during the 2-year follow-up period (108.3 vs. 135.0, respectively). The least squares mean difference (SE) between the first and highest tertiles of total PCBs was 26.7 (18.9) at the 2-year follow-up. Similarly, subjects in the highest tertile of total OCPs had lower HOMA-β values compared with those in the first tertile at the 2-year follow-up.

## Discussion

In this study, children’s exposure to certain POPs such as two OCPs (*trans*-nonachlor and β-HCH) as well as total marker PCBs was found to be associated with decreased values of HOMA-β. A positive association was observed between *trans*-nonachlor and glucose levels. Additionally, HOMA-β values remained low in subjects who had higher concentrations of total PCBs at the 2-year follow-up, suggesting that higher levels of certain POPs in children might be responsible for impaired insulin secretion, which potentially increases the risk of diabetes later in life.

Previous meta-analyses and/or systematic reviews of data from adult populations indicated a risk of developing T2DM after exposure to POPs, which supports our findings. Total PCBs and HCB were found to be associated with incident T2DM, with pooled odds ratios (ORs) of 2.0 (95% CI: 1.13, 3.53) and 1.7 (95% CI: 1.28, 2.27), respectively (Wu et al. 2013). *trans*-Nonachlor, dioxin, dioxin-like chemicals, DDE, DDT, PCBs, and DDD were positively related to T2DM (Taylor et al. 2013). These studies report summarized estimates and use the prevalence of diabetes as an outcome, rather than metabolic biomarkers. Although our results do not necessarily provide the mechanism and/or causal pathways, they suggest that exposure to these chemical compounds might influence insulin secretion in people.

Various studies have been conducted to investigate the possible links between metabolic biomarkers and POP concentrations. Studies focusing on metabolic biomarkers and exposure to POPs have yielded different results depending on population characteristics such as age, sex, or whether subjects live in areas with high exposure levels. Thus, the interpretation of the results of these studies is complicated. A study conducted among Faroese residents, a fishing population, revealed that the fasting insulin level was reduced by –7% (95% CI: –12, 2), and the fasting glucose level increased by 6% (–1, 13%) in nondiabetic subjects (Grandjean et al. 2011). Lee et al. (2007) identified an association between the risk of diabetes and exposure to POPs and found that certain POPs, including oxychlordane, *trans*-nonachlor, and dioxin-like PCBs, were associated with increased HOMA-IR values among the U.S. population with background exposure to POPs.

In this study, we found that the concentrations of certain POPs showed significant negative associations with HOMA-β values, as an estimate of beta-cell function used to assess insulin secretory function. In addition, participants in the second tertile of exposed children had the greatest reduction in the HOMA-β values at the 2-year follow-up. This is consistent with studies suggesting that even low concentrations of POPs can have a greater impact on type 2 diabetes (T2D) than higher concentrations, due to POPs’ nontraditional dose responses and inverted U-shape association with the end point (Lee et al. 2010; Silverstone et al. 2012). We compared HOMA-β values in different countries. Compared with other Asian countries, we observed similar HOMA-β values among Taiwanese children (181.9 ± 119.2 for boys, 203.7 ± 131.8 for girls) (Hung et al. 2006). Concentrations of several POPs were associated positively with glucose levels and negatively with insulin levels, whereas no significant associations with HOMA-IR were observed. Adjustment for BMI and breastfeeding in analyses of the association between POPs and T2DM has been controversial (Jensen et al. 2014; Taylor et al. 2013). Additional analyses excluding these covariates revealed that the associations did not markedly change after adjustment for breastfeeding. After additional adjustment for BMI, significant associations seemed attenuated and to have disappeared, which is consistent with a previous study (Burns et al. 2014).

Recently, a study in healthy children by Jensen et al. (2014) reported an inverse association between POP exposure levels and metabolic parameters, including HOMA-β. Additionally, the insulin level decreased with exposure to PCBs and OCPs in the higher quintiles of healthy Danish children (Jensen et al. 2014). A prospective study conducted among Russian children living in highly contaminated areas revealed that HOMA-IR and insulin levels were reduced in the higher quintiles of certain serum OCPs, such as B-HCH and *p*,*p*´-DDE. The study also reported that serum leptin secretion diminished with increasing quintiles of HCB, β-HCH, and *p*,*p*´-DDE, which may affect insulin sensitivity (Burns et al. 2014). This was consistent with our finding that exposure to POPs might adversely influence metabolic function, although there were some differences in specific biomarkers. Higher serum POP levels were associated with decreased HOMA-β values and insulin levels, but there was no significant effect on HOMA-IR values, suggesting the possibility that early-life exposure to POPs affects insulin secretion in children exposed to background levels.

A study conducted in Korean children demonstrated that impaired insulin secretion, rather than insulin sensitivity, might be a risk factor for T2DM. HOMA-β values were significantly lower in the diabetes group than the non-diabetes group (52.5 ± 46.5 vs. 446.6 ± 202.1, *p* < 0.001) (Park et al. 2016b). Decreasing insulin secretion in childhood might be responsible for the risk of developing diabetes later in life. The mechanism of association between POPs and insulin secretion is not fully understood. De Tata (2014) revealed an association between dioxin toxicity and beta-cell dysfunction, summarizing epidemiological studies. Hectors et al. (2013) also reported that environmental exposure to POPs might act as a metabolic disruptor, which could affect pancreatic beta-cell function and interfere with normal insulin production.

There are some limitations to the present study. First, although we estimated the sample size and statistical power before conducting the research, follow-up losses could have resulted in insufficient statistical power to achieve significance. Second, fish consumption is a key source of POP exposure (Kris-Etherton et al. 2002; Persky et al. 2001; Schwartz et al. 1983), but we could not incorporate this into the model because of the large amount of missing nutritional data. Instead, we performed a subgroup analysis for 85 children to compare the effect sizes. As shown in Table S2, after further adjustment for total calories, the effect size increased, suggesting that the present study may have underestimated the associations. Lifestyle factors such as dietary habits and physical activities, as well as a family history of diabetes, could also be potential confounders. Last, recent studies suggest that POPs are associated with altered puberty timing in both girls and boys due to interference with hormone receptors, resulting in altered reproductive function (Den Hond et al. 2011; Korrick et al. 2011; Windham et al. 2015). In this study, we were not able to assess the association of the majority of participants with puberty staging due to missing data. However, the majority of participants were prepubertal at baseline, and 20% of them had entered puberty by the 2-year follow-up. The association remained similar after an additional adjustment for puberty (data not shown).

This study also has several strengths. For example, few studies have examined the association between exposure to POPs and metabolic biomarkers among children. This study not only provides scientific evidence of this association but also suggests where further research is needed to explore this association. Also, direct measurements in children’s serum, rather than self-answered questionnaires of dietary intakes, might improve the validity of current exposures in children (Hoppin et al. 2000; Karmaus and Zhu 2004). We assessed the associations among metabolic biomarkers, not only for specific compounds but also for mixtures of PCBs and OCPs, by summing individual chemicals to observe the combined toxic effects on the human body, as in other studies (Pan et al. 2009; Schell et al. 2014). This can also be a limitation, because we assumed that a given mixture was additive, disregarding other possible effects, such as synergism or antagonism. Finally, a longitudinal data analysis was conducted based on a 2-year follow-up after baseline to investigate whether exposure to POPs affects subsequent HOMA-β changes.

## Conclusion

In conclusion, we observed that POPs were inversely associated with HOMA-β in children age 7–9 years, whereas no association was found for HOMA-IR. Even though our findings could not conclusively confirm that these environmental chemicals affect insulin sensitivity directly, they suggest a potential mechanism whereby POPs might decrease insulin secretory function. Future studies will address the risk factors for POP exposure in children as well as investigate the possible outcomes using longitudinal data. Studies over extended periods of time are needed to clarify whether high POP exposures affect insulin secretory function and increase the risk of diabetes.

## Supplemental Material

(127 KB) PDF
