# Supplementary material for: Serum Levels of Persistent Organic Pollutants and Insulin Secretion among Children Age 7–9 Years: A Prospective Cohort Study
Source: Environ Health Perspect. 2016 Jun 7;124(12):1924–30. doi: 10.1289/EHP147 (PMC5132629; doi:10.1289/EHP147)
Supplement: (127 KB) PDF [file EHP147.s001.acco.pdf]

Environ Health Perspect
DOI: 10.1289/EHP147

Note to readers with disabilities: *EHP* strives to ensure that all journal content is accessible to all readers. However, some figures and Supplemental Material published in *EHP* articles may not conform to 508 standards due to the complexity of the information being presented. If you need assistance accessing journal content, please contact ehp508@niehs.nih.gov. Our staff will work with you to assess and meet your accessibility needs within 3 working days.

Supplemental Material

Serum Levels of Persistent Organic Pollutants and Insulin Secretion among Children Aged 7 to 9 Years: A Prospective Cohort Study

Su Hyun Park, Eun-Hee Ha, Young Sun Hong, and Hyesook Park

Table of Contents

Table S1. Comparison of study and non-study subjects aged 7 to 9
Table S2. Adjusted percentage change in HOMA-β according to POP concentration tertile (exposure, ng/g lipid) (n=85)

Table S1. Comparison of study and non-study subjects aged 7 to 9

	Study subjects (n=132)	Non-study subjects (n=75)	*p*-value
BMI (kg/m²)	15.80 (15.00-17.50)	16.34 (14.90-17.83)	0.6335
Glucose (mg/dL)	82.00 (78.00-87.00)	81.00 (77.00-84.00)	0.1233
Insulin (μIU/mL)	7.50 (6.09-9.27)	7.83 (6.19-10.09)	0.2673
Triglyceride (mg/dL)	51.00 (36.00-72.00)	56.00 (37.00-80.00)	0.3834
HDL Cholesterol (mg/dL)	62.00 (53.00-69.00)	62.00 (53.00-67.00)	0.9740

Data are expressed as median (IQR). BMI=body mass index; HDL=high density lipoprotein.

Table S2. Adjusted percentage change[a] in HOMA-β according to POP concentration tertile (exposure, ng/g lipid) (n=85)

	Model 1		Model 2	
**Compounds[b,c]**	β	(95% CI)	β	(95% CI)
**PCB 138**				
≤1.76	Referent		Referent	
1.761-3.06	-3.92	(-21.34, 17.35)	-7.69	(-34.30, 29.69)
>3.07	-14.79	(-30.23, 4.08)	-7.69	(-33.63, 28.40)
**PCB 153**				
≤3.05	Referent		Referent	
3.06-6.06	-9.52	(-25.92, 9.42)	-4.88	(-30.23, 29.69)
>6.06	-14.79	(-30.23, 4.08)	-19.75	(-41.73, 10.52)
**PCB 180**				
≤18.73	Referent		Referent	
19.17-31.73	-1.00	(-18.94, 20.92)	-17.30	(-39.95, 12.75)
>31.74	-6.76	(-24.42, 15.03)	-13.06	(-36.24, 18.53)
**Total PCBs[d]**				
≤18.73	Referent		Referent	
19.17-31.73	-8.61	(-24.42, 10.52)	2.02	(-23.66, 36.34)
>31.74	-17.30	(-32.29, 1.01)	-9.52	(-35.60, 27.12)
**Marker PCBs[e]**				
≤9.00	Referent		Referent	
9.05-14.89	1.01	(-16.47, 22.14)	2.02	(-22.12, 34.99)
>14.89	-18.13*	(-32.97, -1.00)	-14.79	(-38.12, 17.35)
**β-HCH**				
≤4.36	Referent		Referent	
4.37-9.00	1.01	(-17.30, 22.14)	-20.55	(-42.88, 10.52)
>9.00	-22.12*	(-36.24, -4.88)	-28.11*	(-47.27, -1.98)
**p,p'-DDE**				
≤31.45	Referent		Referent	
31.46-61.03	-13.93	(-28.82, 5.13)	-21.34	(-44.01, 10.52)
>61.03	-16.47	(-31.61, 1.01)	-10.42	(-36.87, 27.12)
**T-Nonachlor**				
≤0.45	Referent		Referent	
0.90-1.54	-1.98	(-18.94, 18.53)	3.05	(-22.89, 39.10)
>1.54	-25.92**	(-38.74, -10.42)	-27.39	(-47.27, 0.10)
**Total OCPs[f]**				
≤52.54	Referent		Referent	
53.84-96.62	-0.10	(-17.30, 20.92)	11.63	(-21.34, 56.83)
>96.62	-13.93	(-28.82, 4.08)	7.25	(-23.66, 49.18)

HOMA-β, homeostatic model assessment of beta-cell function; PCB, polychlorinated biphenyls; β-HCH, β-hexachlorocyclohexane; HCB, hexachlorobenzene; p,p'-DDE, p,p'-dichlorodiphenyldichloroethylene; T-Nonachlor, trans-nonachlor; OCP, organochlorine pesticides
[a]Adjusted percentage change = [exp(β)-1] × 100 [b]Log transformed [c]The limit of detection was set as LOD/2 [d]Sum of all measured 32 PCBs [e]Sum of marker PCBs (PCB 28,52,101,138,180) [f]Sum of all measured 19 OCPs

Model 1 was adjusted for baseline age (months), sex, mother's education level, ponderal index, breastfeeding (yes/no) and WHO BMI z-score
Model 2 was adjusted for baseline age (months), sex, mother's education level, ponderal index, breastfeeding (yes/no), WHO BMI z-score, and total calories
*p<0.05 **p<0.01
